# Biodiversity of *Borrelia burgdorferi* Strains in Tissues of Lyme Disease Patients

**DOI:** 10.1371/journal.pone.0022926

**Published:** 2011-08-04

**Authors:** Dustin Brisson, Nilofer Baxamusa, Ira Schwartz, Gary P. Wormser

**Affiliations:** 1 Biology Department, University of Pennsylvania, Philadelphia, Pennsylvania, United States of America; 2 Division of Infectious Diseases of the Department of Medicine, New York Medical College, Valhalla, New York, United States of America; 3 Department of Microbiology and Immunology, New York Medical College, Valhalla, New York, United States of America; University of Edinburgh, United Kingdom

## Abstract

Plant and animal biodiversity are essential to ecosystem health and can provide benefits to humans ranging from aesthetics to maintaining air quality. Although the importance of biodiversity to ecology and conservation biology is obvious, such measures have not been applied to strains of an invasive bacterium found in human tissues during infection. In this study, we compared the strain biodiversity of *Borrelia burgdorferi* found in tick populations with that found in skin, blood, synovial fluid or cerebrospinal fluid of Lyme disease patients. The biodiversity of *B. burgdorferi* strains is significantly greater in tick populations than in the skin of patients with erythema migrans. In turn, strains from skin are significantly more diverse than strains at any of the disseminated sites. The cerebrospinal fluid of patients with neurologic Lyme disease harbored the least pathogen biodiversity. These results suggest that human tissues act as niches that can allow entry to or maintain only a subset of the total pathogen population. These data help to explain prior clinical observations on the natural history of *B. burgdorferi* infection and raise several questions that may help to direct future research to better understand the pathogenesis of this infection.

## Introduction

*Borrelia burgdorferi* infection in the United States has a variety of clinical presentations in Lyme disease patients varying from asymptomatic infection to chronic arthritis. The most common clinical manifestation is a characteristic skin lesion, known as erythema migrans, caused by a cutaneous *B. burgdorferi* infection [1]. The natural history of untreated patients with erythema migrans is that approximately 5% of patients will develop carditis (e.g., heart block), approximately 10% will develop neurologic manifestations such as meningitis, cranial nerve palsy or radiculopathy and approximately 60% will develop arthritis [2]. At least 20% of patients do not develop any subsequent clinical manifestations. The variability in clinical manifestations among patients could result from intrinsic differences among people or from biologic differences among the strains of *B. burgdorferi* that initiate the infections.

Strains of *B. burgdorferi* can be classified into subtypes based on various typing methods and increasing evidence suggests that certain subtypes are more likely to cause hematogenous dissemination than others [3]. The aforementioned natural history of untreated patients with erythema migrans suggests the testable hypothesis that the diversity of strains causing cutaneous infection exceeds that of strains causing arthritis and furthermore that the diversity of strains causing arthritis exceeds that which cause neurologic Lyme disease. In this study, we compare the strain biodiversity of *B. burgdorferi* found in ticks, in erythema migrans skin lesions, in blood of patients with erythema migrans, in synovial fluid of patients with Lyme arthritis, and in cerebrospinal fluid (CSF) of patients with neurologic Lyme disease.

## Methods

### Patient populations

To evaluate the diversity of *B. burgdorferi* strains we used previously published data on *ospC* typing of strains present in nymphal tick populations [4], [5], [6], in the skin of patients with erythema migrans [3], in the blood of patients with erythema migrans [3], in the synovial fluid of patients with Lyme arthritis [7] or in the CSF of patients with neurologic Lyme disease [8]. The groups evaluated were identical to those previously reported [3] except that the borrelial isolates recovered from patients with erythema migrans who also had concomitant neurologic manifestations of Lyme disease were excluded from the present analyses. The ticks that were sampled were from Long Island and the Lower Hudson Valley region of New York State [4], [5], [6]. The patients with erythema migrans were individuals from the Lower Hudson Valley who had been enrolled in prospective studies at New York Medical College in Valhalla, NY [3]. The patients with Lyme arthritis were from New England [7]. Of the 16 patients with neurologic Lyme disease included in this analysis, 11 were from New York State, 3 were from Connecticut, 1 was from Pennsylvania, and 1 was from California [8]. The patients with Lyme disease fulfilled the criteria of the Centers for Disease Control and Prevention for the surveillance of Lyme disease [9].

Strains were typed at the *ospC* locus. The *ospC* allele of each strain was determined by PCR followed by reverse line blot or DNA sequencing [10]. *ospC* is highly polymorphic and is in linkage disequilibrium with many other genetic loci in *B. burgdorferi* making it an easily identifiable marker of lineage diversity within populations.

### Statistics

Strain biodiversity of *B. burgdorferi* in tick populations and in human tissue samples was calculated using classical ecological biodiversity methods, including Shannon's diversity index and Simpson's concentration index [11]. The Shannon's and Simpson's indices were compared for sample sets of borrelial strains present in eight nymphal tick populations, human erythema migrans skin lesions, human blood, human synovial fluid and human CSF. Additionally, to determine if the strain diversity found in human blood differed from that found in skin due only to sampling a limited subset of the strains found in the skin, we created 100 datasets by randomly sampling 124 strains, the number of strains analyzed from the blood, from the skin dataset.

Shannon's diversity index, the most commonly used statistic to estimate biodiversity, incorporates both the number and the evenness of categorical types (*ospC* in the present case). Shannon's index (H′) was calculated as H′ = −Σ(p_i_*ln(p_i_))−[(S−1)/2N] where p_i_ is the proportion of type *i*, S is the number of types, and N is the total number of samples. Simpson's index represents the probability that two randomly chosen *B. burgdorferi* strains from a given sample are of the same *ospC* type [12]. The unbiased estimator of the Simpson index is given by (N/(N−1))(1−λ) where λ = Σ(p_i_^2^) and N is the number of observations. This index also accounts for both the number of types present and the relative abundance of each type. Confidence intervals about the mean and statistical differences among samples were assessed by bootstrap resampling.

## Results

We evaluated the *B. burgdorferi* strain biodiversity based on *ospC* type from eight tick populations and from the following tissue sites in human patients: erythema migrans skin lesions, blood of patients with erythema migrans, synovial fluid of patients with Lyme arthritis, and CSF of patients with neurologic Lyme disease. Borrelial strains from blood, skin and CSF were cultured prior to *ospC* typing, while strains in ticks and synovial fluid were assayed directly without prior culture (Table 1). The borrelial strains analyzed were predominantly from New York State, except for the synovial strains which were from New England.

**Table 1:** *ospC* types of *Borrelia burgdorferi* found in tissues of human patients.

	Tick	Skin	Blood	Synovial Fluid	CSF
Study	[4,5,6]	[3]	[3]	[7]	[8]
Material assessed	Direct PCR	Cultured isolates	Cultured isolates	Direct PCR	Cultured isolates
Totals	763	278	124	49	16
A	83	45	30	11	4
B	74	37	18	5	1
C	19	2	2	1	0
D	68	4	0	0	0
E	53	14	1	1	0
F	51	9	2	1	0
G	46	14	1	2	0
H	48	13	7	5	0
I	24	19	15	0	1
J	22	3	1	1	0
K	132	84	39	21	10
M	51	3	1	0	0
N	24	17	7	1	0
O	1	1	0	0	0
T	39	2	0	0	0
U	28	11	0	0	0

**Figure 1:**
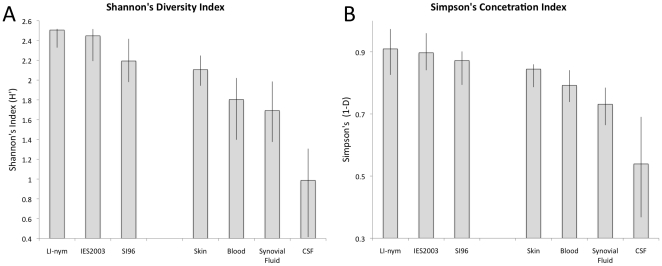
Strain biodiversity of *B. burgdorferi* in tick populations and human tissues using A) Shannon's and B) Simpson's index. Biodiversity is significantly lower in human tissues than in all but one nymphal tick population. The greatest strain biodiversity was found in skin and the least diversity in CSF. However, synovial fluid strains of *B. burgdorferi* were only slightly less diverse than blood strains. These conclusions are supported equally by Shannon's and Simpson's biodiversity index.

Strain biodiversity based on *ospC* typing was significantly greater in all but one of the 8 tick populations evaluated (p<0.05) than in any of the human samples, including skin samples (Figure 1 and Table S1). At least 12 of the 16 *ospC* types previously described in Northeastern *B. burgdorferi* populations [4], [5], [6], [13], [14], [15] were present in all of the tick populations. Every strain present in each of the tick populations was relatively well represented leading to high evenness in the *ospC* frequency distribution and thus high biodiversity values on both Shannon's and Simpson's index.

Fifteen of the 16 *ospC* types were detected at least once in the set of strains isolated from the skin of patients with erythema migrans. However, the frequencies of *ospC* types from human skin was heavily biased toward types A, B, I, K, and N with the remaining types poorly represented (Table 1). Indeed, six of the *ospC* types were represented by fewer than four samples each (1.4%). Thus, despite having similar strain richness (the total number of *ospC* types represented), the biodiversity of strains from erythema migrans skin lesions was significantly lower (p<0.05) than from all but one of the tick populations (p = 0.094) due to the low evenness in the frequency distribution of *ospC* types found in skin.

The strain richness and evenness found in disseminated infections was lower than the diversity found in erythema migrans skin lesions, resulting in significantly lower biodiversity values in the both Shannon's and Simpson's indices (p = 0.023/0.028 for blood; p = 0.015/0.022 for synovial fluid; p<0.001/0.002 for CSF) (Figure 1). Disseminated infections had fewer total types represented (12 in blood, 10 in synovial fluid, and 4 in CSF), and the *B. burgdorferi ospC* types in all three sites were strongly biased toward types A and K. To determine if the strain diversity found in blood was lower than that found in skin due simply to biases associated with sampling a limited subset of the strains found in the skin, we compared the diversity found in blood samples to that of 100 datasets created by randomly sampling a subset of strains from the skin sample set. The strain diversity found in the blood sample was significantly lower than all subsampled datasets for both diversity metrics (p<0.03/0.035). These analyses indicate that the population of strains isolated from patient blood is different than the population of strains isolated from erythema migrans skin lesions.

Among the samples from disseminated sites, samples isolated from blood were the most diverse followed by samples isolated from synovial fluid and CSF. Strain diversity found in blood, however, was not significantly greater than the diversity found in synovial fluid in either Shannon's or Simpson's index (p>0.05). Samples from blood and synovial fluid also retained similar type richness (12 and 10, respectively) and similar biases for types A, B and K. A striking difference between blood and synovial fluid, however, was the inclusion of type I in blood samples (12%) and the complete exclusion of type I from synovial fluid samples (Table 1). Consistent with this observation, an analysis of frequencies demonstrated a significant difference between the frequency distribution of *ospC* types from blood and from synovial fluid strains driven largely by the absence of *ospC* type I in synovial fluid (p<0.05).

Shannon's and Simpson's index values for strains isolated from CSF samples were significantly lower than for the strains isolated from blood (p = 0.013/0.009) or synovial fluid (p = 0.032/0.049). CSF strains of *B. burgdorferi* were represented almost exclusively by types A and K (80% of the strains), with just one B and one I strain. The 95% confidence intervals were much greater for CSF than other tissue sites primarily due to the limited sample size (n = 16).

## Discussion

This study demonstrates that the biodiversity of strains of *B. burgdorferi* – as delineated by the *ospC* allele - is significantly greater in ticks than in the skin of patients with erythema migrans. In turn, skin isolates are significantly more diverse than any of the disseminated sites: blood, synovial fluid and CSF. These results expand upon and further clarify previously reported data and conclusions [3], [7], [8]. Although there were some differences in the geographic origin of the borrelial strains analyzed, for the most part they were from the Northeastern region of the United States. Within this limited geographic area there is no evidence of local variation in the presence or frequency of *ospC* types in tick populations [6], [15], [16]. The conclusions drawn from the present data may not pertain to more distant geographic areas where genotypic variation may occur.

Our findings suggest that although virtually any strain can cause erythema migrans, the distribution of infecting genotypes is biased toward specific *B. burgdorferi* lineages. This implies that *ospC* genotypes define distinct *B. burgdorferi* genomic lineages that possess different, as yet unidentified, virulence properties that influence the likelihood of causing cutaneous infection. An alternative explanation, however, is that the lower diversity in skin was due to a selection bias introduced by *in vitro* culture, since the skin isolates were cultured, while the tick strains were directly identified by PCR.

Strain biodiversity from both blood and synovial fluid is significantly lower than strain biodiversity in erythema migrans skin lesions. Although it could be argued that a limitation of this analysis is that all of the blood isolates considered were from patients who had concomitant erythema migrans, recovery of *B. burgdorferi* from the blood of patients with extracutaneous manifestations of Lyme disease in the absence of erythema migrans is extremely unusual [17]. Importantly, the diversity found in the blood of patients is not a random subset of the skin samples, but is far less diverse due to a strong over-representation of specific *B. burgdorferi* lineages suggesting that biodiversity decreases due to selective barriers to dissemination.

Despite the expectation for a greater barrier to enter the synovial fluid than to enter the blood, strain biodiversities from the synovial fluid and the blood were comparable. This is in agreement with prior observations that both spirochetemia and arthritis may occur in at least 60% of untreated adult patients with erythema migrans [2], [18]. Nevertheless, there were significant differences in the frequency of particular strains between blood and synovial fluid. Thus, hematogenous dissemination alone is sufficient to explain joint involvement in this infection for most, but not all strains of *B. burgdorferi*. Tropism for joints is unusual among bacteria that enter the blood stream, even for other spirochetes [19], and a mechanism to explain this tropism has yet to be identified.

Our findings help explain why neurologic manifestations of Lyme disease are less frequent than joint manifestations among untreated patients with erythema migrans [2]. We hypothesize that either only a small subset of the *B. burgdorferi* strains can persist in the CSF or that the CSF is protected by a barrier that only a limited subset of strains of *B. burgdorferi* can penetrate. Interestingly, Grab et al. [20] demonstrated that *B. burgdorferi* strains of varying *ospC* type had different capacities to cross an *in vitro* model of the blood-brain barrier, implying a mechanism to account for the low biodiversity of pathogens found in CSF samples. Future research should focus on elucidating the particular virulence properties of *B. burgdorferi* that predispose to both blood stream invasion and neurologic involvement.

The pattern of *B. burgdorferi* strain composition in disseminated sites contrasts with a neutral pattern of dissemination within hosts [21]. In a neutral dissemination process, the probability of detecting a *B. burgdorferi* lineage in a disseminated site would equal the frequency of the lineage in ticks or in the skin. Thus, the diversity found at disseminated sites, as measured across a population of hosts, would be the same as the diversity found in tick or skin samples. The contrast between the present data and the neutral expectation indicates that the decrease in diversity in disseminated sites does not result from a random bottleneck process, but from either different likelihoods of dissemination to, or persistence at, the site among lineages.

Although measures of biodiversity are widely used in ecological studies [22], [23], this is the first time that such measures have been applied to strains of an invasive bacterium found in various tissue sites during human infection. We used classical ecological metrics of biodiversity, Shannon's diversity index and Simpson's concentration index, which are commonly used to compare species and population biodiversity of plants and animals. Shannon's index reflects type richness and evenness and is considered the best measure of their combined influence [24]. Simpson's index also accounts for type richness and the relative abundance of each type; the simplicity of its interpretation has resulted in many similar statistics in other fields [25]. Using these classical ecological statistics, we have shown that different human tissues support different levels of pathogen biodiversity in a process that has certain similarities to habitats that support differing plant and animal diversity. For example, biomes and niches with limited resources or low productivity support less diverse plant and animal communities as well as lower population variability [26], [27]. We have now shown that tissues within a host may support varying levels of biodiversity of a pathogen population. These findings help to explain prior clinical observations on the natural history of *B. burgdorferi* infection and raise several questions that may help to direct future research to understand better the pathogenesis of this infection.

## Supporting Information

Table S1 The biodiversity of *B. burgdorferi* types from tick populations and human tissues as estimated by Shannon's Diversity Index and Simpson's Concentration index.
(DOC)
