# Supplementary material for: Biodiversity of Borrelia burgdorferi Strains in Tissues of Lyme Disease Patients
Source: PLoS One. 2011 Aug 4;6(8):e22926. doi: 10.1371/journal.pone.0022926 (PMC3150399; doi:10.1371/journal.pone.0022926)
Supplement: Table S1 — The biodiversity of B. burgdorferi types from tick populations and human tissues as estimated by Shannon's Diversity Index and Simpson's Concentration index. (DOC) [file pone.0022926.s001.doc]

**Table S1**. The biodiversity of *B. burgdorferi* types from tick populations and human tissues as estimated by Shannon’s Diversity Index and Simpson’s Concentration index. Biodiversity *B. burgdorferi* as measured by *ospC* strain diversity is greatest in the tick populations and decreases dramatically in the internal human tissue sites. This pattern is supported by both the Shannon’s Diversity Index and Simpson’s Concentration index. Confidence intervals were estimated by bootstrap resampling of the data.

| Location | Shannon’s Diversity index (95%CI) | Simpson’s Concentration index  (95%CI) |
| --- | --- | --- |
| LI-nymphs | 2.51  (2.3-2.5) | 0.909  (0.83-0.97) |
| Westchester nymphs | 2.46  (2.5-2.3) | 0.900  (0.92-0.86) |
| IES2003 nymphs | 2.45  (2.2-2.52) | 0.897  (0.84-0.96) |
| Shelter Island 1994 nymphs | 2.42  (2.5-2.2) | 0.900  (0.93-0.83­) |
| IES2002 nymphs | 2.35  (2.5-2.2) | 0.886  (0.90-0.84) |
| IES2004 nymphs | 2.31  (2.5-2.1) | 0.885  (0.90-0.82) |
| Wildwood nymphs | 2.28  (2.5-2.1) | 0.881  (0.90-0.82) |
| Shelter Island 1996 nymphs | 2.19  (2.0-2.4) | 0.872  (0.79-0.90) |
| Skin | 2.12  (1.9-2.2) | 0.844  (0.79-0.86) |
| Blood | 1.80  (1.4-2.0) | 0.792  (0.74-0.850 |
| Synovial fluid | 1.69  (1.5-2.0) | 0.741  (0.67-0.80) |
| CSF | 0.99  (0.4-1.3) | 0.539  (0.37-0.69) |
